# Relating cardiorespiratory responses to work rate during incremental ramp exercise on treadmill in children and adolescents: sex and age differences

**DOI:** 10.1007/s00421-021-04741-1

**Published:** 2021-06-18

**Authors:** Nicola Lai, Justin J. Fiutem, Nora Pfaff, Desy Salvadego, James Strainic

**Affiliations:** 1 Department of Mechanical, Chemical and Materials Engineering, University of Cagliari, Cagliari, Italy; 2 Departments of Pediatrics, Case Western Reserve University, Cleveland, OH USA; 3 Biomedical Engineering, Case Western Reserve University, Cleveland, OH USA; 4 Rainbow Babies and Children’s Hospital, Cleveland, OH USA; 5 Department of Pediatrics, University of California, San Francisco, CA USA; 6 Department of Automation, Biocybernetics and Robotics, Jožef Stefan Institute, Ljubljana, Slovenia

**Keywords:** Kinetics, Muscular efficiency, CPET slopes, Oxygen delivery, External work rate

## Abstract

**Purpose:**

Evaluation of cardiopulmonary exercise testing (CPET) slopes such as $d\mathrm{H}\mathrm{R}/d{\mathrm{W}\mathrm{R}}_{\mathrm{t}\mathrm{o}\mathrm{t}}$ (cardiac/skeletal muscle function) and ${d \dot{V}{\text{O}} }_{2}/d{\mathrm{W}\mathrm{R}}_{\mathrm{t}\mathrm{o}\mathrm{t}}$ (O_2_ delivery/utilization), using treadmill protocols is limited because the difficulties in measuring the total work rate (${\mathrm{W}\mathrm{R}}_{\mathrm{t}\mathrm{o}\mathrm{t}}$). To overcome this limitation, we proposed a new method in quantifying ${\mathrm{W}\mathrm{R}}_{\mathrm{t}\mathrm{o}\mathrm{t}}$ to determine CPET slopes.

**Methods:**

CPET’s were performed by healthy patients, (*n* = 674, 9–18 year) 300 female (F) and 374 male (M), using an incremental ramp protocol on a treadmill. For this protocol, a quantitative relationship based on biomechanical principles of human locomotion, was used to quantify the ${\mathrm{W}\mathrm{R}}_{\mathrm{t}\mathrm{o}\mathrm{t}}$ of the subject. CPET slopes were determined by linear regression of the data recorded until the gas exchange threshold occurred.

**Results:**

The method to estimate ${\mathrm{W}\mathrm{R}}_{\mathrm{t}\mathrm{o}\mathrm{t}}$ was substantiated by verifying that: $d{ \dot{V}{\text{O}} }_{2}/d{\mathrm{W}\mathrm{R}}_{\mathrm{t}\mathrm{o}\mathrm{t}}$ for treadmill exercise corresponded to an efficiency of muscular work similar to that of cycle ergometer; $d{ \dot{V}{\text{O}} }_{2}/d{\mathrm{W}\mathrm{R}}_{\mathrm{t}\mathrm{o}\mathrm{t}}$ (mL min^−1^ W^−1^) was invariant with age and greater in M than F older than 12 years old (13–14 years: 9.6 ± 1.5(F) vs. 10.5 ± 1.8(M); 15–16 years: 9.7 ± 1.7(F) vs. 10.6 ± 2.2(M); 17–18 years: 9.6 ± 1.7(F) vs. 11.0 ± 2.3(M), *p* < 0.05); similar to cycle ergometer exercise, $dHR/d{WR}_{tot}$ was inversely related to body weight (BW) (*r* = 0.71) or $\dot{V}{\text{O}}_{{2,{\text{~peak}}}}$ (*r* = 0.66) and $d{ \dot{V}{\text{O}} }_{2}/d{\mathrm{W}\mathrm{R}}_{\mathrm{t}\mathrm{o}\mathrm{t}}$ was not related to BW (*r* = − 0.01), but had a weak relationship with $\dot{V}{\text{O}}_{{2,{\text{~peak}}}}$ (*r* = 0.28).

**Conclusion:**

The proposed approach can be used to estimate ${\mathrm{W}\mathrm{R}}_{\mathrm{t}\mathrm{o}\mathrm{t}}$ and quantify CPET slopes derived from incremental ramp protocols at submaximal exercise intensities using the treadmill, like the cycle ergometer, to infer cardiovascular and metabolic function in both healthy and diseased states.

## Introduction

Cardiopulmonary exercise testing (CPET) continues to be the ideal method in assessing cardiovascular and respiratory function in children and adolescents (Guazzi et al. 2017). CPET is performed using a cycle ergometer or a treadmill (Paridon et al. 2006; Armstrong and Welsman 2019a) with a progressive increase in workload to challenge the subject to the limit of tolerance. The work rate ($\mathrm{W}\mathrm{R}$) can be imposed and measured in protocols with the cycle ergometer in a precise manner, whereas in treadmill CPET, the $\mathrm{W}\mathrm{R}$ depends on body weight, inclination, running speed, and other factors that cannot be easily quantified.

This cycle ergometer feature allows clinicians and researchers to relate pulmonary gas-exchange and heart rate adjustments to work rate by $d{ \dot{V}{\text{O}} }_{2}/d\mathrm{W}\mathrm{R}$ and $d\mathrm{H}\mathrm{R}/d\mathrm{W}\mathrm{R}$ (i.e., CPET slopes) to evaluate cardiorespiratory function and the efficiency of O_2_ delivery and utilization processes (Cooper et al. 2014). CPET slopes quantify the integrated responses of the cardiovascular, respiratory, and skeletal muscle systems to an increase in ATP demand and provide complementary diagnostic information to the maximal pulmonary O_2_ uptake (${ \dot{V}{\text{O}} }_{2,\mathrm{m}\mathrm{a}\mathrm{x}}$). Because CPET slopes are obtained from data recorded at submaximal exercise intensities (Cooper et al. 2014), they represent a suitable alternative to ${ \dot{V}{\text{O}} }_{2,\mathrm{m}\mathrm{a}\mathrm{x}}$ measurement in assessing cardiorespiratory function in pediatric populations for those subjects who cannot reliably reach ${ \dot{V}{\text{O}} }_{2,\mathrm{m}\mathrm{a}\mathrm{x}}$ (Shaibi et al. 2006; Poole and Jones 2017).

The CPET slopes are clinically relevant in both pediatric (Groen et al. 2010) and adult populations (Troutman et al. 1998; Guazzi et al. 2012, 2017; Ashish et al. 2015; Barron et al. 2016; Popovic et al. 2019). Their determinations rely on the knowledge of $\mathrm{W}\mathrm{R}$ also referred to as total work rate (${\mathrm{W}\mathrm{R}}_{\mathrm{t}\mathrm{o}\mathrm{t}}$) in this manuscript and can only be determined when using cycle ergometer protocols. In treadmill protocols, especially in clinical settings, it is difficult to quantify ${\mathrm{W}\mathrm{R}}_{\mathrm{t}\mathrm{o}\mathrm{t}}$. The possibility of relating CPET variables to $WR$ could enable investigators to analyze CPET slopes obtained from treadmill protocols.

Computational and experimental studies provide robust information on quantifying the external (${\mathrm{W}\mathrm{R}}_{\mathrm{e}\mathrm{x}\mathrm{t}}$) and internal work rate as major components of the work rate for walking and running (Cavagna and Kaneko 1977; Willems et al. 1995; Minetti 2000). Specifically, studies on kinetic movements showed that the ${\mathrm{W}\mathrm{R}}_{\mathrm{e}\mathrm{x}\mathrm{t}}$ fraction was approximately 40–50% and 65–75% of the ${\mathrm{W}\mathrm{R}}_{\mathrm{t}\mathrm{o}\mathrm{t}}$ (internal and external) for walking and running, respectively (Minetti et al. 1994b, 2000; Willems et al. 1995). This relationship can quantify ${\mathrm{W}\mathrm{R}}_{\mathrm{t}\mathrm{o}\mathrm{t}}$ from ${\mathrm{W}\mathrm{R}}_{\mathrm{e}\mathrm{x}\mathrm{t}}$ which could be estimated from biomechanical principles of human locomotion. This approach to estimate ${\mathrm{W}\mathrm{R}}_{\mathrm{t}\mathrm{o}\mathrm{t}}$ for treadmill protocols is promising but has not been tested yet.

Physiological functions change over the course of growth and development (Cooper et al. 1984, 2014) and CPET slopes are a valuable tool to evaluate these changes. In a cycle ergometer study (Cooper et al. 2014) for children and adolescents, $d\mathrm{W}\mathrm{R}/d\mathrm{H}\mathrm{R}$ and $d{ \dot{V}{\text{O}} }_{2}/d\mathrm{H}\mathrm{R}$ slopes were linearly related to body size whereas $d{ \dot{V}{\text{O}} }_{2}/d\mathrm{W}\mathrm{R}$ slope was body weight independent. In the same study, these slopes have been reported to be lower in females than males. These CPET slope characteristics (muscular efficiency, sex differences, dependence on body weight) observed for cycle ergometer are expected to be similar for treadmill protocols and can be used to verify the consistency of the ${\mathrm{W}\mathrm{R}}_{\mathrm{t}\mathrm{o}\mathrm{t}}$ estimate for treadmill exercise.

The aim of this study was to develop an equation to quantify ${\mathrm{W}\mathrm{R}}_{\mathrm{t}\mathrm{o}\mathrm{t}}$ for incremental ramp protocols using a treadmill and provide evidence that ${\mathrm{W}\mathrm{R}}_{\mathrm{t}\mathrm{o}\mathrm{t}}$ estimates are consistent with CPET slope characteristics. The equation is based on the relationship between ${\mathrm{W}\mathrm{R}}_{\mathrm{t}\mathrm{o}\mathrm{t}}$ and ${\mathrm{W}\mathrm{R}}_{\mathrm{e}\mathrm{x}\mathrm{t}}$. ${\mathrm{W}\mathrm{R}}_{\mathrm{e}\mathrm{x}\mathrm{t}}$ is estimated from the work required to move an object in a plane at a specific inclination and speed (Margaria 1978; Porszasz et al. 2003). To determine the efficacy of the method proposed to estimate ${\mathrm{W}\mathrm{R}}_{\mathrm{t}\mathrm{o}\mathrm{t}}$, we evaluate whether (a) $d{ \dot{V}{\text{O}} }_{2}/d{\mathrm{W}\mathrm{R}}_{\mathrm{t}\mathrm{o}\mathrm{t}}$ for treadmill was similar to the efficiency of muscular work for walking, running and cycling (Whipp and Wasserman 1969; Brooks 2012) and (b) the CPET slopes ($d\mathrm{H}\mathrm{R}/d{\mathrm{W}\mathrm{R}}_{\mathrm{t}\mathrm{o}\mathrm{t}}$ and $d{ \dot{V}{\text{O}} }_{2}/d{\mathrm{W}\mathrm{R}}_{\mathrm{t}\mathrm{o}\mathrm{t}}$) are related to body weight or ${ \dot{V}{\text{O}} }_{2,\mathrm{p}\mathrm{e}\mathrm{a}\mathrm{k}}$. Also, we substantiate the versatility of the ${\mathrm{W}\mathrm{R}}_{\mathrm{t}\mathrm{o}\mathrm{t}}$ equation by investigating gender and age differences in the CPET slopes obtained in children and adolescents exercising on a treadmill. We hypothesize that in adolescents: (1) males have a higher $d{ \dot{V}{\text{O}} }_{2}/d{\mathrm{W}\mathrm{R}}_{\mathrm{t}\mathrm{o}\mathrm{t}}$ and $d{ \dot{V}{\text{O}} }_{2}/d\mathrm{H}\mathrm{R}$ than females; (2) females have higher $d\mathrm{H}\mathrm{R}/d{\mathrm{W}\mathrm{R}}_{\mathrm{t}\mathrm{o}\mathrm{t}}$ than males; and (3) $d\mathrm{H}\mathrm{R}/d{\mathrm{W}\mathrm{R}}_{\mathrm{t}\mathrm{o}\mathrm{t}}$ decrease and $d{ \dot{V}{\text{O}} }_{2}/d\mathrm{H}\mathrm{R}$ increase with age; (4) CPET slopes are related to body weight except $d{ \dot{V}{\text{O}} }_{2}/d{\mathrm{W}\mathrm{R}}_{\mathrm{t}\mathrm{o}\mathrm{t}}$.

## Methods

This was a retrospective study based on data from 1934 patients, mainly children and adolescents who had undergone CPET on a treadmill at Rainbow Babies and Children’s Hospital between the years 2000 and 2013. The study initially included healthy subjects and patients with congenital heart disease (ages 9–18 years); the analysis focused on the healthy patients. Patients were excluded if they were underweight with a BMI less than the 5^th^ percentile, overweight with a BMI greater than the 85^th^ percentile, had cardiopulmonary disease, had an RER < 1.0 or an exhaustion time less than 350 s. The final number of included patients in the study was 674 (300 females and 374 males).

**Fig. 1:**
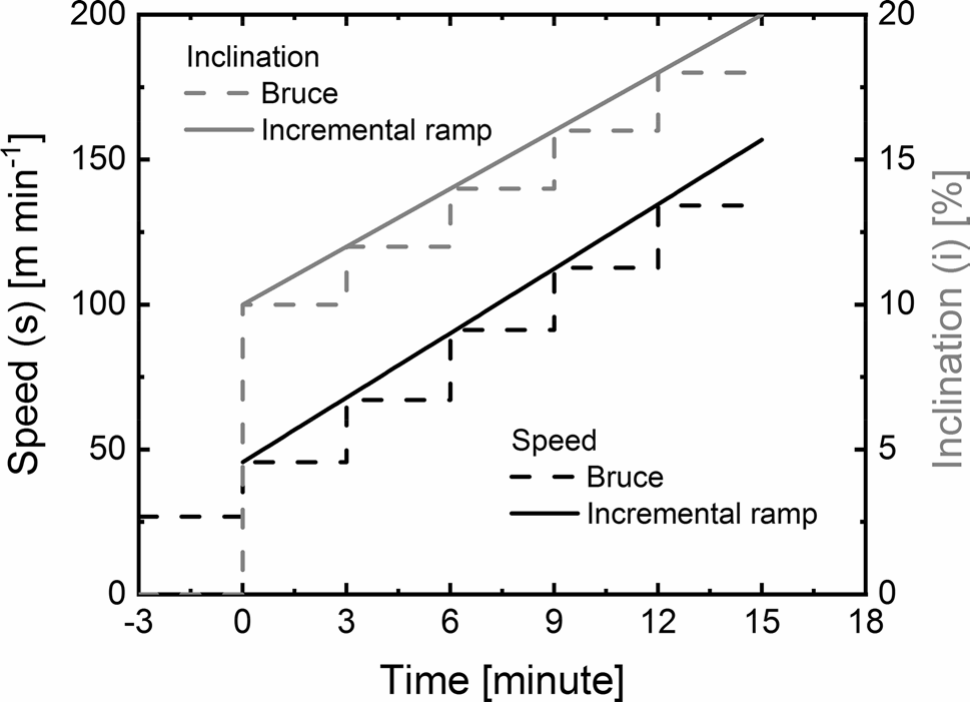
Comparison between incremental ramp protocol used in this study and Bruce protocol: speed and inclination changes with time

The subjects underwent a modified Bruce protocol with an incremental ramp (DiBella et al. 2002) with a linear change in both speed and slope of the treadmill (Fig. 1). During the test, a 12-lead EKG was used to measure heart rate ($\mathrm{H}\mathrm{R}$), while the metabolic cart was used to determine pulmonary oxygen uptake (${ \dot{V}{\text{O}} }_{2}$), carbon dioxide release (${\dot{\mathrm{V}\mathrm{C}\mathrm{O}}}_{2}$), and minute ventilation (${\dot{\mathrm{V}}}_{\mathrm{e}}$).

The protocol consisted of three phases: warmup, exercise, and recovery. The test started with 3 min of walking at 26.8 m min^−1^ (warm-up) and 0% incline and continued until speed and inclination were increased to 45.6 m min^−1^ and 10%, respectively. After 3 min, both speed ($s$) and inclination ($i$) were increased every minute by 7.4 m min^−1^ and 0.66%, respectively. Thus, $s$ and $i$ change with time ($t$) according to the relationships:

1 $$s\left( t \right) = 45.6 + 7.4\;t.$$

2 $$i\left(t\right)=10+0.66\;t.$$

The subjects continued exercise until they reached the limit of tolerance.

The external work rate (${\mathrm{W}\mathrm{R}}_{\mathrm{e}\mathrm{x}\mathrm{t}}$) generated by the subject running on the treadmill with an angle of inclination $\alpha (t)$ and speed $s\left(t\right)$ was estimated as:

3 $${\text{WR}}_{{{\text{ext}}}} (t) = m\;g\;s\left( t \right)\;{\text{sin~}}\left[ {\alpha (t)} \right].$$

where $m$ and $g$ represent the body mass and the acceleration of gravity, respectively. The angle of inclination is related to the inclination by the trigonometric relationship:

4 $${\text{sin}}\;\left[ {\alpha (t)} \right] = i\left( t \right)/\sqrt {1 + i\left( t \right)^{2} } .$$

It was assumed that the fraction of the external work rate (${f}_{\mathrm{e}\mathrm{x}\mathrm{t}}$) was the same at different speeds and inclinations. The external work rate was 70% of the total work rate (${f}_{\mathrm{e}\mathrm{x}\mathrm{t}}=0.7$) (Gosseye et al. 2010). With this assumption, the total work rate can be computed as a function of ${\mathrm{W}\mathrm{R}}_{\mathrm{e}\mathrm{x}\mathrm{t}}$, and ${f}_{\mathrm{e}\mathrm{x}\mathrm{t}}$:

5 $${\mathrm{W}\mathrm{R}}_{\mathrm{t}\mathrm{o}\mathrm{t}}(t)={\mathrm{W}\mathrm{R}}_{\mathrm{e}\mathrm{x}\mathrm{t}}(t)/{f}_{\mathrm{e}\mathrm{x}\mathrm{t}}.$$

The estimate of ${\mathrm{W}\mathrm{R}}_{\mathrm{t}\mathrm{o}\mathrm{t}}$ by Eq. 5 accounts for inclination, speed, and body weight of the subject running on the treadmill. The data were processed using excel macros. Outliers sporadically observed in HR and breath-by-breath CPET data were identified and excluded for the determination of peak (i.e., ${ \dot{V}{\text{O}} }_{2}$ and ${\mathrm{W}\mathrm{R}}_{\mathrm{t}\mathrm{o}\mathrm{t}}$) or slopes.

A facemask (8900 Series, Hans Rudolph, Inc.; Kansas, MO) was carefully fitted and sealed with a gel (Hans Rudolph, Inc.) to obviate any gas leaks during exercise. The subjects were given several minutes to familiarize themselves with the breathing apparatus to minimize unusual breathing patterns. To measure gas exchange, subjects breathed through a mass flow sensor (hot-wire anemometer) connected to a metabolic cart system (VMax 29, SensorMedics, Yorba Linda, CA). Before each exercise test, the volume sensor was calibrated using a 3-L syringe while the O_2_ and CO_2_ analyzers were calibrated as previously reported (Lai et al. 2008).

Gas exchange threshold (GET, ${ \dot{V}{\text{O}} }_{2,\mathrm{G}\mathrm{E}\mathrm{T}}$) was determined by the V-slope method in combination with ventilatory equivalents and the end-tidal O_2_ responses (Beaver et al. 1986). Peak values for $\mathrm{H}\mathrm{R}$, ${ \dot{V}{\text{O}} }_{2}$, ${\dot{\mathrm{V}\mathrm{C}\mathrm{O}}}_{2}$, O_2_ pulse calculated as ${ \dot{V}{\text{O}} }_{2}/\mathrm{H}\mathrm{R}$, and ${\dot{\mathrm{V}}}_{\mathrm{e}}$ were determined as the average of the last 20 s prior to exhaustion. Data were processed with a moving average of 10 s.

For each subject’s data, a linear regression between ${\mathrm{W}\mathrm{R}}_{\mathrm{t}\mathrm{o}\mathrm{t}}$ and time, ${ \dot{V}{\text{O}} }_{2}$ and time, $\mathrm{H}\mathrm{R}$ and ${\mathrm{W}\mathrm{R}}_{\mathrm{t}\mathrm{o}\mathrm{t}}$, ${ \dot{V}{\text{O}} }_{2}$ and $\mathrm{H}\mathrm{R}$, ${ \dot{V}{\text{O}} }_{2}$ and ${\mathrm{W}\mathrm{R}}_{\mathrm{t}\mathrm{o}\mathrm{t}}$ was obtained to determine the slopes for $d{\mathrm{W}\mathrm{R}}_{\mathrm{t}\mathrm{o}\mathrm{t}}/dt$, $d{ \dot{V}{\text{O}} }_{2}/d\mathrm{t}$ $d\mathrm{H}\mathrm{R}/d{\mathrm{W}\mathrm{R}}_{\mathrm{t}\mathrm{o}\mathrm{t}}$, $d{ \dot{V}{\text{O}} }_{2}/d\mathrm{H}\mathrm{R}$, and $d{ \dot{V}{\text{O}} }_{2}/d{\mathrm{W}\mathrm{R}}_{\mathrm{t}\mathrm{o}\mathrm{t}}$. The linear regression was performed with the recorded data in the interval of time between 60 s after the onset of exercise until GET occurred.

### Statistical analysis

The results are reported as means ± standard deviation. Linear regression was used to evaluate the relationship between CPET slopes and body weight and between CPET slopes and ${ \dot{V}{\text{O}} }_{2,\mathrm{p}\mathrm{e}\mathrm{a}\mathrm{k}}$. The correlation between the variable was quantified with Pearson’s coefficient (*r*). The comparisons of the CPET variables and slopes obtained from female and male tests for different age groups were evaluated with a two-way ANOVA with Bonferroni for multiple comparisons (Origin software). The interaction effect between age and gender was investigated for the CPET slopes. Student *t*-test was used to compare regression lines between female and male groups and determine whether the parameters of the regression lines were significantly different. Difference of *p* < 0.05 was considered significant.

## Results

Anthropometric measurements were grouped by sex and age and reported in Table 1. Both height and weight increased with age and there were no significant differences in height, weight, or BMI percentiles between male and female patients. For both female and male groups, BMI percentile was similar at 50–60%.

**Table 1:** Summary of the anthropometric measurement of the population

	9–10	11–12	13–14	15–16	17–18
N. Subjects (–)					
Female	19	39	86	121	35
Male	26	66	106	107	69
Height (m)					
Female	1.42 ± 0.08	1.52 ± 0.07^§^	1.60 ± 0.07†	1.64 ± 0.06†	1.64 ± 0.07†
Male	1.39 ± 0.08	1.41 ± 0.08^§^	1.68 ± 0.09*,†	1.74 ± 0.08*,†	1.76 ± 0.06*,†
Weight (kg)					
Female	36.1 ± 6.1	45.7 ± 7.5§	52.6 ± 8.3†	59.0 ± 8.7†	61.2 ± 9.6†
Male	35.2 ± 5.8	42.5 ± 7.5§	59.0 ± 11.1*,†	66.6 ± 9.8*,†	71.8 ± 9.5*,†
BMI (kg m^−2^)					
Female	17.9 ± 2.1	19.7 ± 2.2	20.3 ± 2.5^§^	21.7 ± 2.7†	22.7 ± 3.2†
Male	18.0 ± 2.0	18.4 ± 2.1	20.7 ± 2.6†	21.8 ± 2.4†	23.1 ± 2.4†
BMI (Percentile)					
Female	58.2 ± 24.9	64.5 ± 20.7	55.4 ± 24.8	58.8 ± 24.5	59.0 ± 26.0
Male	62.1 ± 26.4	53.5 ± 25.6	61.9 ± 25.6	60.7 ± 24.2	60.3 ± 23.8

*Influence of gender: statistically different from female (*p* < 10^–3^)

^†^Influence of age within the group: statistically different from 9- to 10- and 11–12-year-old groups (*p* < 10^–2^)

^§^Influence of age within the group: statistically different from 9- to 10-year-old group (*p* < 10^–4^)

### Cardiopulmonary response to peak exercise: sex and age

CPET variables for males and females measured among different age groups are reported in Table 2. Maximal HR was not affected by sex, whereas ${ \dot{V}{\text{O}} }_{2,\mathrm{G}\mathrm{E}\mathrm{T}}$, O_2_ pulse, peak of ${\mathrm{W}\mathrm{R}}_{\mathrm{t}\mathrm{o}\mathrm{t}}$, ${\dot{\mathrm{V}}}_{\mathrm{e}}$, and ${ \dot{V}{\text{O}} }_{2}$ were higher in male than in female patients > 12 years of age. The peak ${\mathrm{W}\mathrm{R}}_{\mathrm{t}\mathrm{o}\mathrm{t}}$ and O_2_ pulse increased with age in both sexes.

**Table 2:** Summary of the CPET variables measured during the incremental ramp protocol

	9–10	11–12	13–14	15–16	17–18
Exercise time (min)					
Female	7.3 ± 1.1	7.4 ± 1.2	7.9 ± 1.1	8.1 ± 1.4	7.9 ± 1.6
Male	7.8 ± 0.9	8.5 ± 1.4*	9.2 ± 1.2*,†	9.7 ± 1.4*,†	9.7 ± 1.3*,†
Peak HR (beat min^−1^)					
Female	192 ± 10	193 ± 11	193 ± 11	190 ± 10	190 ± 12
Male	192 ± 10	191 ± 9	194 ± 9	191 ± 10	188 ± 11
Peak ${\dot{\mathbf{V}\mathbf{O}}}_{2}$ (mL kg^−1^ min^−1^)					
Female	41.5 ± 6	40.0 ± 7	41.7 ± 6	41.3 ± 7	39.8 ± 7
Male	47.9 ± 8	48.5 ± 6*	51.0 ± 7*,†	52.5 ± 8*,†	52.7 ± 8*,†
Peak ${\dot{\mathbf{V}\mathbf{C}\mathbf{O}}}_{2}$ (mL kg^−1^ min^−1^)					
Female	45.8 ± 7	45.1 ± 9	47.7 ± 7	48.3 ± 8	47.0 ± 9
Male	50.2 ± 8	52.6 ± 7*	56.7 ± 7*,†	59.3 ± 8*,†	59.8 ± 8*,†
Pea*k *$\boldsymbol{R}\boldsymbol{E}\boldsymbol{R}$ (–)					
Female	1.10 ± 0.05	1.12 ± 0.07	1.14 ± 0.06	1.17 ± 0.08	1.18 ± 0.07
Male	1.05 ± 0.04	1.08 ± 0.06	1.11 ± 0.06	1.13 ± 0.07*	1.14 ± 0.08
Peak ${\dot{\boldsymbol{V}}}_{\mathbf{e}}$ (mL kg^−1^ min^−1^)					
Female	1.60 ± 0.4	1.48 ± 0.3	1.47 ± 0.3	1.45 ± 0.3	1.38 ± 0.2
Male	1.62 ± 0.3	1.62 ± 0.4	1.74 ± 0.3*	1.73 ± 0.3*	1.69 ± 0.3*
Peak ${\dot{\mathbf{V}\mathbf{O}}}_{2}/\mathbf{H}\mathbf{R}$ (mL beat^−1^)					
Female	7.8 ± 1.7	9.4 ± 1.9	11.3 ± 2.2^§^	12.8 ± 2.7†	12.7 ± 2.6†
Male	8.7 ± 1.3	10.7 ± 2.2	15.5 ± 3.5*,†	18.3 ± 3.7*,†	20.1 ± 3.9*,†
${\dot{\mathbf{V}\mathbf{O}}}_{2,\mathbf{G}\mathbf{E}\mathbf{T}}$ (mL kg^−1^ min^−1^)					
Female	29.4 ± 4	28.9 ± 5	29.7 ± 5	29.8 ± 5	28.6 ± 5
Male	33.6 ± 5	34.9 ± 5*	35.3 ± 5*	35.9 ± 6*	35.9 ± 6*
Peak ${\mathbf{W}\mathbf{R}}_{\mathbf{t}\mathbf{o}\mathbf{t}}$ (W)					
Female	126 ± 28	160 ± 30	194 ± 38†	224 ± 46†	225 ± 44†
Male	129 ± 22	167 ± 40^§^	252 ± 60*,†	299 ± 57*,†	322 ± 57*,†

*Influence of gender: statistically different from female (*p* < 10^–3^)

^†^Influence of age within the group: statistically different from 9- to 10- and 11–12-year-old groups (*p* < 10^–2^)

^§^Influence of age within the group: statistically different from 9- to 10-year-old group (*p* < 10^–4^)

### CPET slopes at submaximal exercise: relating the estimate of ${\mathbf{W}\mathbf{R}}_{\mathbf{t}\mathbf{o}\mathbf{t}}$ to ${\dot{\mathbf{V}\mathbf{O}}}_{2}$ and $\mathbf{H}\mathbf{R}$

**Fig. 2:**
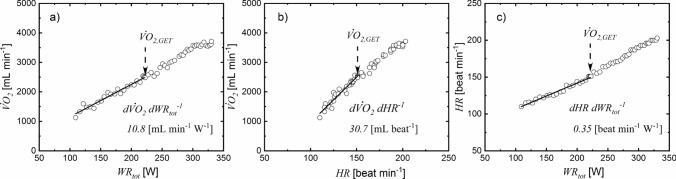
Representative case to determine the slope of $d{ \dot{V}{\text{O}} }_{2}/d{\mathrm{W}\mathrm{R}}_{\mathrm{t}\mathrm{o}\mathrm{t}}$ (**a**), $d{ \dot{V}{\text{O}} }_{2}/d\mathrm{H}\mathrm{R}$ (**b**), and $d\mathrm{H}\mathrm{R}/d{\mathrm{W}\mathrm{R}}_{\mathrm{t}\mathrm{o}\mathrm{t}}$ (**c**) by linear regression

**Fig. 3:**
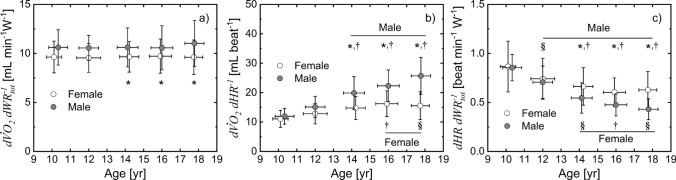
Effects of gender and age on the slope $d{\dot{VO}}_{2}/d{WR}_{tot}$ (**a**), $d{ \dot{V}{\text{O}} }_{2}/d\mathrm{H}\mathrm{R}$ (**b**), $d\mathrm{H}\mathrm{R}/d{\mathrm{W}\mathrm{R}}_{\mathrm{t}\mathrm{o}\mathrm{t}}$ **(c**). ^*^Influence of gender: statistically different from female for $d{ \dot{V}{\text{O}} }_{2}/d{\mathrm{W}\mathrm{R}}_{\mathrm{t}\mathrm{o}\mathrm{t}}$ (*p* < 0.05) and for $d{ \dot{V}{\text{O}} }_{2}/d\mathrm{H}\mathrm{R}$ and $d\mathrm{H}\mathrm{R}/d{\mathrm{W}\mathrm{R}}_{\mathrm{t}\mathrm{o}\mathrm{t}}$ (*p* < 10^–4^). †Influence of age: within the group: statistically different from 9–10 and 11–12 years old groups (*p* < 10^–3^). ^§^Influence of age within the group: statistically different from 9–10 years old (*p* < 10^–3^)

The $d{ \dot{V}{\text{O}} }_{2}/d{\mathrm{W}\mathrm{R}}_{\mathrm{t}\mathrm{o}\mathrm{t}}$, $d{ \dot{V}{\text{O}} }_{2}/\mathrm{d}\mathrm{H}\mathrm{R}$, and $d\mathrm{H}\mathrm{R}/d{\mathrm{W}\mathrm{R}}_{\mathrm{t}\mathrm{o}\mathrm{t}}$ individual slopes were determined using data recorded before the gas exchange threshold, as reported in Fig. 2. The range of $d{ \dot{V}{\text{O}} }_{2}/d{\mathrm{W}\mathrm{R}}_{\mathrm{t}\mathrm{o}\mathrm{t}}$ slopes (Fig. 3a, 9.5–11 mL O_2_ min^−1^ W^−1^) based on the estimate of ${\mathrm{W}\mathrm{R}}_{\mathrm{t}\mathrm{o}\mathrm{t}}(t)$ across different age groups corresponded to a range of typical values of muscular efficiency from 30 to 26% (assuming an energy equivalent for oxygen of 20.9 J mlO_2_^−1^). The $d{ \dot{V}{\text{O}} }_{2}/d{\mathrm{W}\mathrm{R}}_{\mathrm{t}\mathrm{o}\mathrm{t}}$ slope was greater in male than in female patients > 12 years of age (Fig. 3a), and the effect of age was absent in both female and male groups. For both $d{ \dot{V}{\text{O}} }_{2}/d\mathrm{H}\mathrm{R}$ and $d\mathrm{H}\mathrm{R}/d{\mathrm{W}\mathrm{R}}_{\mathrm{t}\mathrm{o}\mathrm{t}}$ slopes, there was a significant difference between females and males among different age groups (Fig. 3b, c): $d{ \dot{V}{\text{O}} }_{2}/d\mathrm{H}\mathrm{R}$ was lower and $d\mathrm{H}\mathrm{R}/d{\mathrm{W}\mathrm{R}}_{\mathrm{t}\mathrm{o}\mathrm{t}}$ was higher in females than males. Furthermore, for both males and females $d{ \dot{V}{\text{O}} }_{2}/d\mathrm{H}\mathrm{R}$ increased, whereas $d\mathrm{H}\mathrm{R}/d{\mathrm{W}\mathrm{R}}_{\mathrm{t}\mathrm{o}\mathrm{t}}$ decreased in older subjects. The interaction between sex and age was also statistically significant for both $d{ \dot{V}{\text{O}} }_{2}/d\mathrm{H}\mathrm{R}$ and $d\mathrm{H}\mathrm{R}/d{\mathrm{W}\mathrm{R}}_{\mathrm{t}\mathrm{o}\mathrm{t}}$ slopes.

In Table 3 was reported the total work rate variation with time during the incremental ramp ($d{\mathrm{W}\mathrm{R}}_{\mathrm{t}\mathrm{o}\mathrm{t}}/dt$). The $d{\mathrm{W}\mathrm{R}}_{\mathrm{t}\mathrm{o}\mathrm{t}}/dt$ slope increased with age in both females and males and was higher in males than females in groups older than 12 years old.

**Table 3:** Summary of the CPET variables measured during the incremental ramp protocol

	9–10	11–12	13–14	15–16	17–18
$$\boldsymbol{d}{\mathbf{W}\mathbf{R}}_{\mathbf{t}\mathbf{o}\mathbf{t}}/\boldsymbol{d}\boldsymbol{t}$$ (W min^−1^)					
Female	11.4 ± 1.0	14.4 ± 2.3^§^	16.7 ± 2.6†	18.8 ± 2.8†	19.4 ± 2.8†
Male	11.2 ± 1.8	13.7 ± 2.3^§^	19.5 ± 3.7*,†	22.1 ± 3.1*,†	23.8 ± 3.0*,†
${\dot{\boldsymbol{d}\boldsymbol{V}\mathbf{O}}}_{2}/\boldsymbol{d}{\mathbf{W}\mathbf{R}}_{\mathbf{t}\mathbf{o}\mathbf{t}}$ (mL min^−1^ W^−1^)					
Female	9.6 ± 1.6	9.5 ± 1.5	9.6 ± 1.5	9.7 ± 1.7	9.6 ± 1.7
Male	10.6 ± 1.8	10.6 ± 1.2	10.5 ± 1.8*	10.6 ± 2.2*	11.0 ± 2.3*

*Influence of gender: statistically different from female (*p* < 10^–2^)

^†^Influence of age within the group: statistically different from 9- to 10- and 11–12-year-old groups (*p* < 10^–3^)

^§^Influence of age within the group: statistically different from 9- to 10-year-old group (*p* < 10^–2^)

**Fig. 4:**
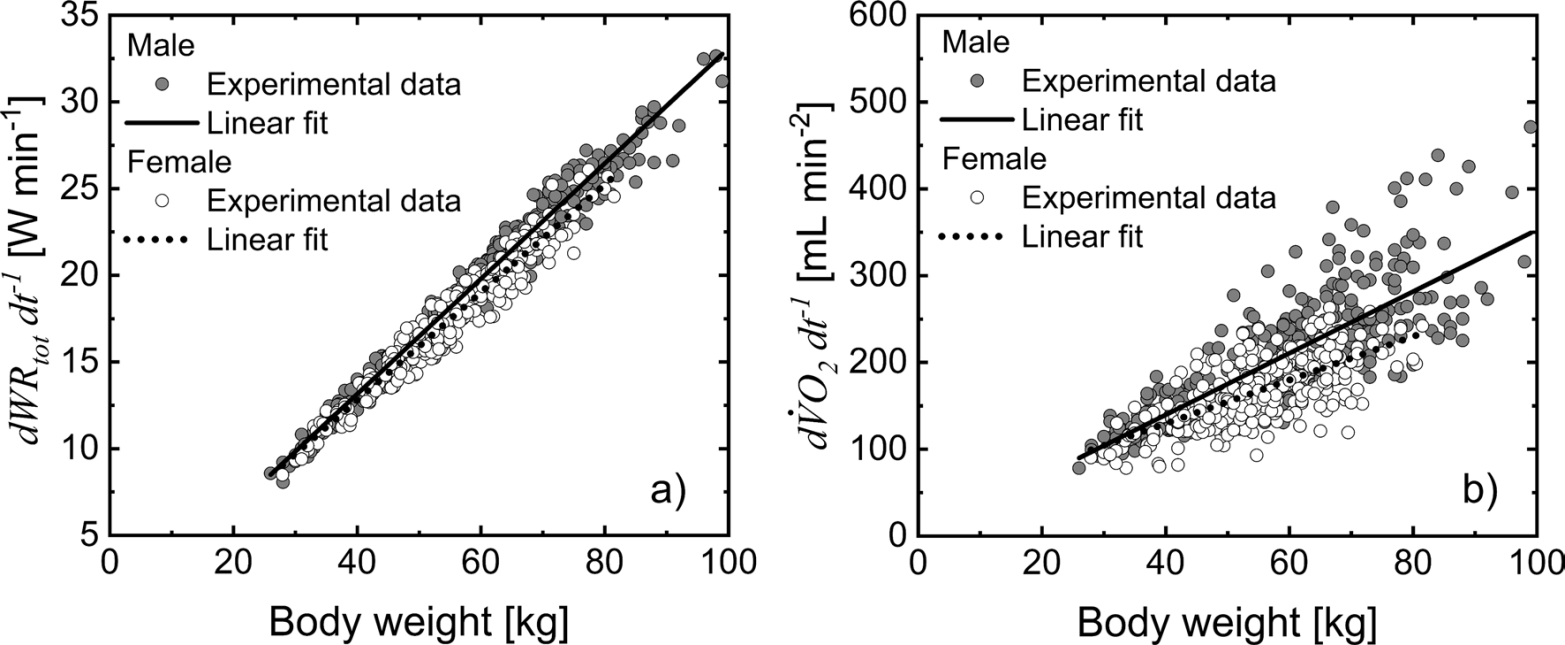
Comparison of the linear relationship $d{\mathrm{W}\mathrm{R}}_{\mathrm{t}\mathrm{o}\mathrm{t}}/dt$ vs. body weight ($BW$) (**a**) between male and female groups, slope is significantly different from zero for male ($d{\text{WR}}_{{{\text{tot}}}} /dt = 0.33{\text{~BW}} - 0.14$, *p* < 10^–8^, solid line) and female ($d{\text{WR}}_{{{\text{tot}}}} /dt = 0.31{\text{~BW}} - 0.37$, *p* < 10^–8^, dot line). Slopes are statistically different *p* < 10^–4^; comparison of the linear relationship $d{ \dot{V}{\text{O}} }_{2}/dt$ vs. $\mathrm{B}\mathrm{W}$ between male and female groups, slope is significantly different from zero for male ($d\dot{V}{\text{O}}_{2} /dt = 3.6{\text{~BW}} - 2.4$, *p* < 10^–8^, solid line) and female ($d\dot{V}{\text{O}}_{2} /dt = 2.5{\text{~BW}} + 29$, *p* < 10^–8^, dot line), slopes are statistically different *p* < 10^–5^

To further investigate the sex differences observed in $d{ \dot{V}{\text{O}} }_{2}/d{\mathrm{W}\mathrm{R}}_{\mathrm{t}\mathrm{o}\mathrm{t}}$, its components $d{\mathrm{W}\mathrm{R}}_{\mathrm{t}\mathrm{o}\mathrm{t}}/dt$ and $d{ \dot{V}{\text{O}} }_{2}/dt$ were analyzed separately. Specifically, $d{\mathrm{W}\mathrm{R}}_{\mathrm{t}\mathrm{o}\mathrm{t}}/dt$ (Fig. 4a) and $d{ \dot{V}{\text{O}} }_{2}/dt$ (Fig. 4b) were plotted against body weight for both male and female groups. Both ${\mathrm{W}\mathrm{R}}_{\mathrm{t}\mathrm{o}\mathrm{t}}/dt$ and $d{ \dot{V}{\text{O}} }_{2}/dt$ have a strong linear relation to body weight. In males, the $d{\mathrm{W}\mathrm{R}}_{\mathrm{t}\mathrm{o}\mathrm{t}}/dt$ change with body weight was only 6% greater than that observed for females, whereas the $d{ \dot{V}{\text{O}} }_{2}/dt$ change with body weight was 41% greater than that observed for females. Thus, the higher $d{ \dot{V}{\text{O}} }_{2}/d{\mathrm{W}\mathrm{R}}_{\mathrm{t}\mathrm{o}\mathrm{t}}$ slopes observed in males is mainly due to the higher $d{ \dot{V}{\text{O}} }_{2}/dt$ because $d{\mathrm{W}\mathrm{R}}_{\mathrm{t}\mathrm{o}\mathrm{t}}/dt$ (i.e., workload changes) in females and males with the same body weight was similar (Fig. 4a).

**Fig. 5:**
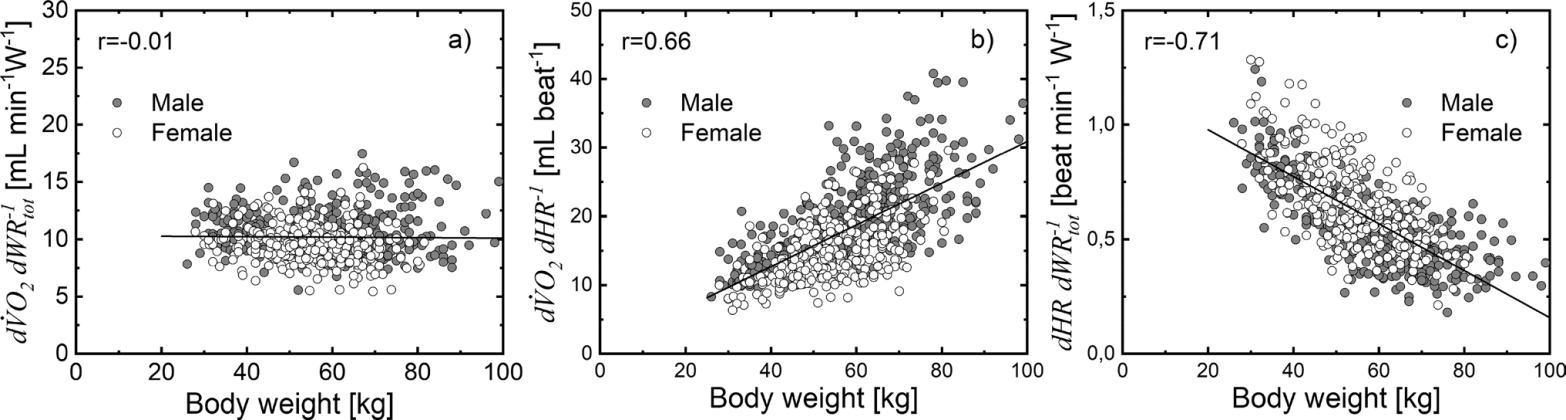
Assessment of the linear relationship between $d{ \dot{V}{\text{O}} }_{2}/d{\mathrm{W}\mathrm{R}}_{\mathrm{t}\mathrm{o}\mathrm{t}}$ and body weight ($BW$) (**a**), slope is not significantly different from zero (*p* > 0.8); $d{ \dot{V}{\text{O}} }_{2}/d\mathrm{H}\mathrm{R}$ and $\mathrm{B}\mathrm{W}$ (**b**), slope significantly different from zero (*p* < 10^–10^); and $dHR/d{WR}_{tot}$ and $BW$ (**c**), slope significantly different from zero (*p* < 10^–10^)

**Fig. 6:**
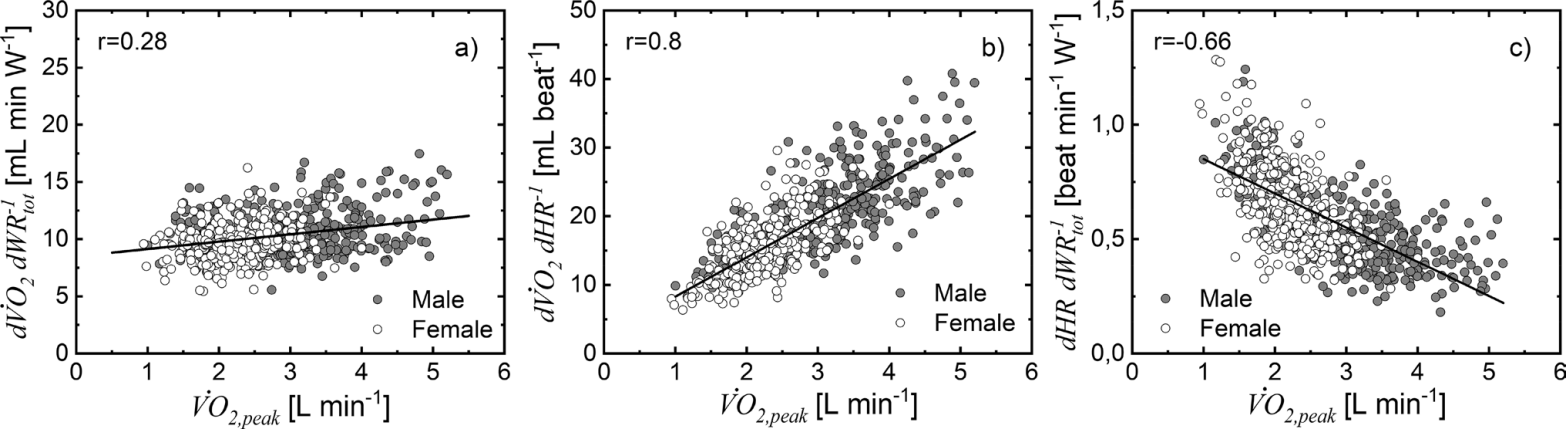
Assessment of the linear regression between $d{ \dot{V}{\text{O}} }_{2}/d{\mathrm{W}\mathrm{R}}_{\mathrm{t}\mathrm{o}\mathrm{t}}$ and ${ \dot{V}{\text{O}} }_{2,\mathrm{p}\mathrm{e}\mathrm{a}\mathrm{k}}$ (**a**); $d{ \dot{V}{\text{O}} }_{2}/d\mathrm{H}\mathrm{R}$ and ${ \dot{V}{\text{O}} }_{2,\mathrm{p}\mathrm{e}\mathrm{a}\mathrm{k}}$ (**b**); $d\mathrm{H}\mathrm{R}/d{\mathrm{W}\mathrm{R}}_{\mathrm{t}\mathrm{o}\mathrm{t}}$ and ${ \dot{V}{\text{O}} }_{2,\mathrm{p}\mathrm{e}\mathrm{a}\mathrm{k}}$ (**c**), slope is significantly different from zero for each case (*p* < 10^–10^)

To further evaluate the reliability of ${\mathrm{W}\mathrm{R}}_{\mathrm{t}\mathrm{o}\mathrm{t}}(t)$ estimates, we tested whether any of the $d{ \dot{V}{\text{O}} }_{2}/d{\mathrm{W}\mathrm{R}}_{\mathrm{t}\mathrm{o}\mathrm{t}}$, $d{ \dot{V}{\text{O}} }_{2}/d\mathrm{H}\mathrm{R}$, and $d\mathrm{H}\mathrm{R}/d{\mathrm{W}\mathrm{R}}_{\mathrm{t}\mathrm{o}\mathrm{t}}$ slopes were related to body weight (Fig. 5) or $\dot{V}{\text{O}}_{{2,{\text{~peak}}}}$ (Fig. 6). The $d{ \dot{V}{\text{O}} }_{2}/d{\mathrm{W}\mathrm{R}}_{\mathrm{t}\mathrm{o}\mathrm{t}}$ was not related to body weight (Fig. 5a) but it was significantly related to $\dot{V}{\text{O}}_{{2,{\text{~peak}}}}$ (Fig. 6a). The $d{ \dot{V}{\text{O}} }_{2}/d\mathrm{H}\mathrm{R}$ was linearly related to body weight (Fig. 5b) and $\dot{V}{\text{O}}_{{2,{\text{~peak}}}}$ (Fig. 6b), whereas $d\mathrm{H}\mathrm{R}/d{\mathrm{W}\mathrm{R}}_{\mathrm{t}\mathrm{o}\mathrm{t}}$ was inversely related to body weight (Fig. 5c) and $\dot{V}{\text{O}}_{{2,{\text{~peak}}}}$ (Fig. 6c).

## Discussion

This study focused on a method to quantify ${\mathrm{W}\mathrm{R}}_{\mathrm{t}\mathrm{o}\mathrm{t}}$ during incremental ramp exercise on a treadmill in children and adolescents and to evaluate whether the CPET slope characteristics determined with ${\mathrm{W}\mathrm{R}}_{\mathrm{t}\mathrm{o}\mathrm{t}}$ expression are like those obtained with the cycle ergometer. The estimates of ${\mathrm{W}\mathrm{R}}_{\mathrm{t}\mathrm{o}\mathrm{t}}$ for treadmill exercise were consistent with typical values of muscular efficiency equivalent to the $d{ \dot{V}{\text{O}} }_{2}/d{\mathrm{W}\mathrm{R}}_{\mathrm{t}\mathrm{o}\mathrm{t}}$ range values (9.5–11 mL O_2_ min^−1^ W^−1^) observed for both female and male groups. The $d{ \dot{V}{\text{O}} }_{2}/d{\mathrm{W}\mathrm{R}}_{\mathrm{t}\mathrm{o}\mathrm{t}}$ slope is greater in males than females and unchanged with age as previously reported for cycling exercise (Cooper et al. 2014). The proposed equation is also substantiated by the linear relationship between $d\mathrm{H}\mathrm{R}/d{\mathrm{W}\mathrm{R}}_{\mathrm{t}\mathrm{o}\mathrm{t}}$ and body weight or ${ \dot{V}{\text{O}} }_{2,\mathrm{p}\mathrm{e}\mathrm{a}\mathrm{k}}$, and between $d{ \dot{V}{\text{O}} }_{2}/d{\mathrm{W}\mathrm{R}}_{\mathrm{t}\mathrm{o}\mathrm{t}}$ and ${ \dot{V}{\text{O}} }_{2,\mathrm{p}\mathrm{e}\mathrm{a}\mathrm{k}}$ which is similar to that observed using the cycle ergometer (Cooper et al. 2014). Males rely on a higher stroke volume (i.e., $d{ \dot{V}{\text{O}} }_{2}/d\mathrm{H}\mathrm{R}$) than females, whereas females compensate for this difference with larger changes in heart rate for the same workload increase (i.e., $d\mathrm{H}\mathrm{R}/d{\mathrm{W}\mathrm{R}}_{\mathrm{t}\mathrm{o}\mathrm{t}}$). In both males and females, the $d\mathrm{H}\mathrm{R}/d{\mathrm{W}\mathrm{R}}_{\mathrm{t}\mathrm{o}\mathrm{t}}$ decrease and $d{ \dot{V}{\text{O}} }_{2}/d\mathrm{H}\mathrm{R}$ increase with age indicates a compensatory mechanism that was likely due to an increase of stroke volume.

### Maximal work rate

The estimate of the peak ${\mathrm{W}\mathrm{R}}_{\mathrm{t}\mathrm{o}\mathrm{t}}$ observed in this study increases with age as does $\dot{V}{\text{O}}_{{2,{\text{~peak}}}}$ (Table 2). In this study, $\dot{V}{\text{O}}_{{2,{\text{~peak}}}}$ and peak ${\mathrm{W}\mathrm{R}}_{\mathrm{t}\mathrm{o}\mathrm{t}}$ obtained during incremental exercise on a treadmill are 10% and 25% higher than those obtained by (Cooper et al. 2014) incremental exercise on a cycle ergometer in populations with comparable age and body weight. Although these comparisons are limited by the fact that the cycle ergometer and treadmill data are not obtained from the same adolescent groups, the treadmill data are consistently higher than those of the cycle ergometer. The cycle ergometer involves less muscle mass recruitment when compared to the exercise on a treadmill (Krahenbuhl et al. 1985; Armstrong and Welsman 1994) which involves weight bearing muscle groups. In a study with children under the age of 10, $\dot{V}{\text{O}}_{{2,{\text{~peak}}}}$ on a treadmill was 11% higher than that obtained on the cycle ergometer for both females and males (Turley et al. 1995). In another study on children, adolescents, and adults, a similar $\dot{V}{\text{O}}_{{2,{\text{~peak}}}}$ difference was observed in female adolescents (Bar-Yoseph et al. 2019). Thus, a higher peak ${\mathrm{W}\mathrm{R}}_{\mathrm{t}\mathrm{o}\mathrm{t}}$ for treadmill exercise than that for a cycle ergometer is consistent with the $\dot{V}{\text{O}}_{{2,{\text{~peak}}}}$ differences observed between the two exercise modalities. The ${\mathrm{W}\mathrm{R}}_{\mathrm{t}\mathrm{o}\mathrm{t}}$ expression allows for the detection of maximal ${\mathrm{W}\mathrm{R}}_{\mathrm{t}\mathrm{o}\mathrm{t}}$ differences related to sex and age like those for $\dot{V}{\text{O}}_{{2,{\text{~peak}}}}$.

### External and total work rate in relation to ${\dot{\mathbf{V}\mathbf{O}}}_{2}$

The equation used to estimate ${\mathrm{W}\mathrm{R}}_{\mathrm{t}\mathrm{o}\mathrm{t}}$ is based on biomechanical principles to study internal, external, and total work rates in human movement, (Cavagna et al. 1964; Cavagna and Kaneko 1977; Willems et al. 1995; Minetti et al. 2000) and used to optimize treadmill protocols (Porszasz et al. 2003). The external work rate quantifies the power required to move the body on a treadmill at specific speeds and inclines, but it does not include the work made by the joints to move the body’s center of mass (i.e., internal) (Herman 2008). Thus, we related the external to the total work rate for running based on the analysis of the kinetic and gravitational potential energy of the body’s center of mass (Cavagna et al. 1964; Cavagna and Kaneko 1977; Willems et al. 1995; Gosseye et al. 2010). Specifically, it is assumed that the fraction of the external work rate (${f}_{\mathrm{e}\mathrm{x}\mathrm{t}}$) is 70% of the total work rate during the incremental ramp protocol. The value of ${f}_{\mathrm{e}\mathrm{x}\mathrm{t}}$ is close to the range of values (60–75%) previously determined on a treadmill with a plate force to measure the force exerted under the foot during running (Schepens et al. 2001; Gosseye et al. 2010). A similar range was also observed in other studies (Minetti et al. 1994b, 2000; Willems et al. 1995; Saibene and Minetti 2003). Although the assumption of considering ${f}_{\mathrm{e}\mathrm{x}\mathrm{t}}$=70% is consistent with the CPET slope characteristics, it might not fully reflect the individual body movement characteristics at different running speeds. In pediatric and adult studies on locomotion, ${f}_{\mathrm{e}\mathrm{x}\mathrm{t}}$ decreased from 75 to 70% (Minetti et al. 1994b; Schepens et al. 2001; Gosseye et al. 2010) for an increase in running speed in the range (50–85 m min^−1^) used in our protocol. If these ${f}_{\mathrm{e}\mathrm{x}\mathrm{t}}$ changes with speed are considered, $d{ \dot{V}{\text{O}} }_{2}/d{\mathrm{W}\mathrm{R}}_{\mathrm{t}\mathrm{o}\mathrm{t}}$ slope decreases by 10% of the values obtained with a constant ${f}_{\mathrm{e}\mathrm{x}\mathrm{t}}$ (Figs. 2, 3). This potential effect on $d{ \dot{V}{\text{O}} }_{2}/d{\mathrm{W}\mathrm{R}}_{\mathrm{t}\mathrm{o}\mathrm{t}}$ is the same in female and male because the workload was the same for subjects of both sexes with the same body weight (Fig. 4a), thus sex differences observed in our work were not affected. Also, age-related changes in walking characteristics have been reported in children (Malloggi et al. 2019). Potential differences of body movement associated to age, sex, speed and inclination (Minetti et al. 1994a) are not considered in the expression proposed to quantify ${\mathrm{W}\mathrm{R}}_{\mathrm{t}\mathrm{o}\mathrm{t}}$. Nevertheless, the $d{ \dot{V}{\text{O}} }_{2}/d{\mathrm{W}\mathrm{R}}_{\mathrm{t}\mathrm{o}\mathrm{t}}$ (9.5–11 mL min^−1^ W^−1^) values obtained in our study are similar to those obtained for cycle ergometer exercise (Lai et al. 2012; Jones and Poole 2013; Cooper et al. 2014), thus indirectly supporting the validity of the proposed equation to estimate the total work rate. The $d{ \dot{V}{\text{O}} }_{2}/d{\mathrm{W}\mathrm{R}}_{\mathrm{t}\mathrm{o}\mathrm{t}}$ values observed among age groups correspond to the range of muscular work efficiency (30–26%) determined for cycling exercise (Whipp and Wasserman 1969). This evidence indicates that the ${\mathrm{W}\mathrm{R}}_{\mathrm{t}\mathrm{o}\mathrm{t}}$ estimated for males and females at different age groups is consistent with the thermodynamic constraints of the biochemical processes in contracting muscle.

To further substantiate the approach proposed in calculating ${\mathrm{W}\mathrm{R}}_{\mathrm{t}\mathrm{o}\mathrm{t}}$ and estimating CPET slopes at submaximal treadmill exercise, we verify that the relationship between CPET slopes and body weight or ${ \dot{V}{\text{O}} }_{2,\mathrm{p}\mathrm{e}\mathrm{a}\mathrm{k}}$ for the treadmill is similar to that observed for the cycle ergometer (Figs. 5 and 6). The inverse correlation between $d\mathrm{H}\mathrm{R}/{d\mathrm{W}\mathrm{R}}_{\mathrm{t}\mathrm{o}\mathrm{t}}$ and body weight and between $d\mathrm{H}\mathrm{R}/{d\mathrm{W}\mathrm{R}}_{\mathrm{t}\mathrm{o}\mathrm{t}}$ and ${ \dot{V}{\text{O}} }_{2,\mathrm{p}\mathrm{e}\mathrm{a}\mathrm{k}}$ are consistent with the linear relationship between ${d\mathrm{W}\mathrm{R}}_{\mathrm{t}\mathrm{o}\mathrm{t}}/d\mathrm{H}\mathrm{R}$ and body weight and between ${d\mathrm{W}\mathrm{R}}_{\mathrm{t}\mathrm{o}\mathrm{t}}/d\mathrm{H}\mathrm{R}$ and ${ \dot{V}{\text{O}} }_{2,\mathrm{p}\mathrm{e}\mathrm{a}\mathrm{k}}$ observed in cycle ergometer exercise (Cooper et al. 2014). Although $d{ \dot{V}{\text{O}} }_{2}/{d\mathrm{W}\mathrm{R}}_{\mathrm{t}\mathrm{o}\mathrm{t}}$ was not related to body weight, it was significantly related to ${ \dot{V}{\text{O}} }_{2,\mathrm{p}\mathrm{e}\mathrm{a}\mathrm{k}}$ (Fig. 5). Both findings echoed those reported for cycle ergometer exercise in the same age groups. Thus, the ${\mathrm{W}\mathrm{R}}_{\mathrm{t}\mathrm{o}\mathrm{t}}$ estimated using the treadmill protocol is body size dependent and enables the analysis of size-dependent and size-independent properties of the CPET slopes similarly to the analysis obtained using cycle ergometer (Cooper et al. 2014).

### $\boldsymbol{d}\mathbf{H}\mathbf{R}/\boldsymbol{d}{\mathbf{W}\mathbf{R}}_{\mathbf{t}\mathbf{o}\mathbf{t}}$ and $\boldsymbol{d}{\dot{\mathbf{V}\mathbf{O}}}_{2}/\boldsymbol{d}\mathbf{H}\mathbf{R}$: sex and age differences

The higher $d\mathrm{H}\mathrm{R}/d{\mathrm{W}\mathrm{R}}_{\mathrm{t}\mathrm{o}\mathrm{t}}$ and lower $d{ \dot{V}{\text{O}} }_{2}/d\mathrm{H}\mathrm{R}$ slopes in females suggests a sex difference in the cardiovascular response to submaximal exercise (Fig. 3). Whereas the first slope quantifies $\mathrm{H}\mathrm{R}$ change with increasing power output, the second slope quantifies the product of stroke volume and arterio-venous difference. Maximal cardiac output differences between females and males have been reported at different ages (Miyamura and Honda 1973). Specifically, in pediatric populations, males appear to have higher maximal cardiac outputs than females and this difference in part is related to a higher stroke volume in males (Rowland et al. 2000). Also, at submaximal exercise intensities, males relied on higher stroke volume with a lower HR (Turley and Wilmore 1997a, b; Armstrong et al. 2007) whereas arterio-venous oxygen difference was similar between genders. In these studies, the greater stroke volume observed in males was mainly attributed to a larger left ventricular muscle size (Turley and Wilmore 1997b). Thus, in our study, under the assumption that the arterio-venous oxygen difference in male is like that in female, the greater $d{ \dot{V}{\text{O}} }_{2}/d\mathrm{H}\mathrm{R}$ in male patients was consistent with the finding of a higher stroke volume in males as compared to female patients. Furthermore, the higher $d\mathrm{H}\mathrm{R}/d{\mathrm{W}\mathrm{R}}_{\mathrm{t}\mathrm{o}\mathrm{t}}$ in females (Fig. 3c) was consistent with a higher amplitude of $\mathrm{H}\mathrm{R}$ per $\mathrm{W}\mathrm{R}$ observed at moderate and heavy exercise intensities in females (Lai et al. 2016). In another study relating CPET variables to work rate for treadmill exercise (Bar-Yoseph et al. 2019), the $d\mathrm{H}\mathrm{R}/d\mathrm{W}\mathrm{R}$ slope in children and adolescents was similar to that found in our study. In this study, $\mathrm{W}\mathrm{R}$ was determined imposing metabolic efficiency, whereas in our work, $\mathrm{W}\mathrm{R}$ was independently derived from a biomechanical relationship between external and total work rate.

An age effect on the slopes was also observed in our study. The increase of $d{ \dot{V}{\text{O}} }_{2}/d\mathrm{H}\mathrm{R}$ with age is in part related to the growth of left ventricular mass contributing to higher stroke volume (Daniels et al. 1995). It has been previously reported that the stroke volume of older children is higher than that of younger children for a given exercise intensity (Bar-Or 1983). Thus, the decrease of $d\mathrm{H}\mathrm{R}/d{\mathrm{W}\mathrm{R}}_{\mathrm{t}\mathrm{o}\mathrm{t}}$ with age observed in our study is consistent with a compensatory mechanism of an increase in stroke volume (indicated by an increase in the $d{ \dot{V}{\text{O}} }_{2}/d\mathrm{H}\mathrm{R}$ slope). This cardiovascular adaptation in exercise is known in the pediatric population (Bar-Or 1983). Sex differences for $d{ \dot{V}{\text{O}} }_{2}/d\mathrm{H}\mathrm{R}$ during growth as observed in our study in subjects older than 12 years old, can be partially explained by a greater growth in left ventricular mass in males when compared to females undergoing pubertal development (Pelà et al. 2016).

Our analysis was based on retrospective data obtained from exercise tests in which lean body mass was not systematically recorded. The analysis was mainly focused on corroborating the method proposed to estimate ${\mathrm{W}\mathrm{R}}_{\mathrm{t}\mathrm{o}\mathrm{t}}$. Although we cannot quantify the effects of body composition on CPET slopes, we can exclude any effect of BMI on sex differences because BMI was comparable in males and females at each age group (Cooper et al. 2016). To study sex differences in ${ \dot{V}{\text{O}} }_{2,\mathrm{p}\mathrm{e}\mathrm{a}\mathrm{k}}$ during development it is crucial to account for lean body mass (Armstrong and Welsman 2019b). Our work focused on CPET slopes, which in the case of $dHR/d{WR}_{tot}$ and $d{ \dot{V}{\text{O}} }_{2}/d\mathrm{H}\mathrm{R}$, should also be affected by body composition.

In conclusion, the results of this work indicate that the methodology proposed has the potential to be used as a tool in clinical and research settings to estimate the workload to determine the CPET slopes derived from an incremental ramp protocol using the treadmill. The estimates of the ${\mathrm{W}\mathrm{R}}_{\mathrm{t}\mathrm{o}\mathrm{t}}$ for treadmill were substantiated by verifying thermodynamic constraint and CPET slope characteristics. Nevertheless, future work should focus on further validating the proposed approach. A direct comparison of treadmill and cycle ergometer exercises as well as ways to change and measure work rate from individual locomotion characteristics for treadmill exercise can contribute to the design of validation strategies. As for the cycle ergometer, CPET slopes depending on ${\mathrm{W}\mathrm{R}}_{\mathrm{t}\mathrm{o}\mathrm{t}}$, can be used to infer cardiovascular and metabolic function at submaximal exercise intensities in healthy and diseased states.

## List of symbols

$d\mathrm{H}\mathrm{R}/d{\mathrm{W}\mathrm{R}}_{\mathrm{t}\mathrm{o}\mathrm{t}}$: Slope: variation of $\mathrm{H}\mathrm{R}$ per variation of ${\mathrm{W}\mathrm{R}}_{\mathrm{t}\mathrm{o}\mathrm{t}}$
$d{ \dot{V}{\text{O}} }_{2}/d\mathrm{H}\mathrm{R}$: Slope: variation of ${ \dot{V}{\text{O}} }_{2}$ per variation of $\mathrm{H}\mathrm{R}$
$d{ \dot{V}{\text{O}} }_{2}/d{\mathrm{W}\mathrm{R}}_{\mathrm{t}\mathrm{o}\mathrm{t}}$: Slope: variation of ${ \dot{V}{\text{O}} }_{2}$ per variation of ${\mathrm{W}\mathrm{R}}_{\mathrm{t}\mathrm{o}\mathrm{t}}$
${f}_{\mathrm{e}\mathrm{x}\mathrm{t}}$: Fraction of the external work rate
$g$: Acceleration of gravity
$\mathrm{G}\mathrm{E}\mathrm{T}$: Gas exchange threshold
$$\mathrm{H}\mathrm{R}$$: Heart beat
$$i$$: Inclination
$$m$$: Body mass
$s$: Speed of the subject
$$t$$: Time
${\dot{\mathrm{V}\mathrm{C}\mathrm{O}}}_{2}$: Rate of carbon dioxide release
$${\dot{\mathrm{V}}}_{\mathrm{e}}$$: Ventilation rate
${ \dot{V}{\text{O}} }_{2}$: Pulmonary oxygen uptake
${ \dot{V}{\text{O}} }_{2,\mathrm{G}\mathrm{E}\mathrm{T}}$: Pulmonary oxygen uptake at GET
$\dot{V}{\text{O}}_{{2,{\text{~peak}}}}$: Pulmonary oxygen uptake at peak
${\mathrm{W}\mathrm{R}}_{\mathrm{e}\mathrm{x}\mathrm{t}}$: External work rate
${\mathrm{W}\mathrm{R}}_{\mathrm{t}\mathrm{o}\mathrm{t}}$: Total work rate
